# The structure of the *Gemella haemolysans* M26 IgA1 protease trypsin-like domain

**DOI:** 10.1107/S2053230X25001219

**Published:** 2025-02-28

**Authors:** Norman Tran, Jasmina S. Redzic, Elan Z. Eisenmesser, Todd Holyoak

**Affiliations:** a Department of Biology University of Waterloo Waterloo ONN2L 3G1 Canada; b Department of Biochemistry and Molecular Genetics, School of Medicine University of Colorado Denver Aurora CO80045 USA; University of Leipzig, Germany

**Keywords:** IgA proteases, serine proteases, trypsin, chymotrypsin, M26 proteases

## Abstract

Immunoglobulin A (IgA) proteases are a group of bacterial-derived enzymes that selectivity hydrolyze human IgA in the hinge region that is unique to this immunoglobulin. Several IgA protease (IgAP) families have evolved this ability using both metalloprotease and serine protease chemical mechanisms. One family of metal-dependent IgAPs is the M26 family. This family can be grouped into two subfamilies based upon the presence or absence of a trypsin-like domain found N-terminal to the IgAP domain. The role of this domain in IgAP structure and function is poorly understood. Here, we present the first structural characterization of an M26 IgAP trypsin-like domain from *Gemella haemo­lysans* (*Gh*Trp). These structural data demonstrate that the *Gh*Trp domain possesses a trypsin-like fold but contains significant deviations in the surface-loop structure that is known to be coupled to protease selectivity. The lack of observable catalytic function coupled with the structural data suggest that this domain may exist in a pro-enzyme-like state that can potentially be activated when the domain is N-terminally proteolytically excised from the larger M26 IgAP structure.

## Abstract 2

The 1.75 Å resolution structure of the *G. haemolysans* M26 IgA1 protease trypsin-like domain is presented. The structural data suggest that the domain exists in an inactive pro-enzyme-like state when in the context of the full-length protein. This putative pro-enzyme may be activated after being N-terminally excised from the larger M26 enzyme structure through the potential stabilization of its S1 pocket and rearrangement of adjacent surface loops.

## 1. Introduction

Immunoglobulin A proteases (IgAPs) represent an interesting group of proteolytic enzymes that have convergently evolved to specifically cleave the unique hinge region present in IgA1 from humans and great apes via several different chemical mechanisms. Representative members of all three known IgAP families have been biochemically and structurally characterized. These include an S6 serine IgAP, two M26 metal-dependent IgAPs and most recently an M64 metal-dependent IgAP (Johnson *et al.*, 2009 ▸; Wang *et al.*, 2020 ▸; Redzic *et al.*, 2022 ▸; Tran *et al.*, 2024 ▸).

The M26 IgAP family can be split into two subfamilies with distinct domain architectures. The subfamily represented by the *Gemella haemolysans* IgAP (*Gh*IgAP) contains an additional trypsin-like domain (*Gh*Trp) found N-terminal to the IgAP domain (Supplementary Fig. S1*a*; residues 684–896^1^; Redzic *et al.*, 2022 ▸). This trypsin-like domain is missing from the other subfamily represented by the *Streptococcus pneumoniae* IgAP (Redzic *et al.*, 2022 ▸). Prior studies that compared *GhIgAP* constructs with and without this domain concluded that *Gh*Trp had no effect on IgA1 proteolysis (Redzic *et al.*, 2022 ▸). This left the role of *Gh*Trp in the context of the larger M26 IgAP structure open to further investigation (Redzic *et al.*, 2022 ▸). To gain insight into the structure and potential functional role of this domain, we solved the crystal structure of *Gh*Trp and demonstrated that the domain does indeed possess a trypsin-like protease fold. This fold, however, contains many unique changes in the well characterized surface loops that are known to contribute to trypsin-like protease specificity. The crystal structure suggests that *Gh*Trp, as it exists in the full-length M26 *Gh*IgAP, may be an inactive pro-enzyme. We propose a mechanism of pro-enzyme activation through the proteolytic removal of the N-terminal region of the full-length enzyme from the *Gh*Trp domain.

## 2. Materials and methods

### 2.1. Protein expression and purification

The trypsin-like domain of *G. haemolysans* IgAP (WP_040464465.1; residues 684–896; *Gh*Trp) was cloned into pET-21b with an N-terminal His-tag and thrombin cleavage site as described previously (Redzic *et al.*, 2022 ▸). *Escherichia coli* BL21(DE3) cells were transformed with this vector and used for recombinant protein expression. An overnight culture was inoculated into ZYP-5052 autoinduction medium (Studier, 2005 ▸) at a ratio of 50 ml overnight culture to 1 l final medium volume with a minimum headspace:medium ratio of 1:1. ZYP-5052 medium was supplemented with 50 µg ml^−1^ kanamycin and the cells were grown at 20°C at 150 rev min^−1^ for 40–48 h, harvested at 6000*g* and the cell pellets were stored at −80°C.

All purification steps were carried out at 4°C. Cell pellets were thawed in buffer *A* (25 m*M* HEPES pH 7.5, 0.5 *M* NaCl, 10 m*M* imidazole), passed twice through a French pressure cell (Thermo Fisher Scientific, Waltham, Massachusetts, USA) at 7.6 MPa for cell lysis and debris was removed via high-speed centrifugation at 17 000*g*. The clarified cell lysate was then incubated with Ni–NTA resin (Qiagen) pre-equilibrated in buffer *A* for 1 h. The resin was first washed with ten column volumes (CV) of buffer *B* [25 m*M* HEPES pH 7.5, 0.1%(*v*/*v*) IGEPAL CA-630, 10 m*M* imidazole] to remove nonspecific hydrophobically bound contaminants, followed by a wash with 15 CV buffer *A*. The protein was eluted with buffer *C* (25 m*M* HEPES pH 7.5, 0.5 *M* NaCl, 300 m*M* imidazole). The Ni–NTA flowthrough was concentrated to less than 1 ml and loaded onto a pre-packed HiLoad Superdex 75 pg 16/600 column pre-equilibrated in crystallization buffer (25 m*M* HEPES pH 7.5) and run at 0.5 ml min^−1^. The purity of the protein in the non-aggregate absorbance peak was qualitatively analysed using SDS–PAGE. Pure fractions were concentrated, frozen in pellets by direct immersion in liquid nitrogen and stored at −80°C. Protein concentration was measured using a 1% mass extinction coefficient of 10.95, theoretically determined from the primary sequence of the protein (Gasteiger *et al.*, 2005 ▸).

### 2.2. Protein crystallization

2.0 µl 10 mg ml^−1^*Gh*Trp was mixed with 2.0 µl reservoir solution [0.2 *M* KNO_3_, 22%(*w*/*v*) PEG 3350] in a hanging-drop crystallization tray. Thin plate clusters appeared after several days and were manually manipulated to acquire single crystals suitable for diffraction. Crystals were cryoprotected in 0.2 *M* KNO_3_, 25%(*w*/*v*) PEG 3350 supplemented with 20%(*v*/*v*) PEG 400 before being plunged into liquid nitrogen for data collection.

### 2.3. Data collection and processing

Diffraction data were collected on the CMCF-BM beamline at the Canadian Light Source (CLS) using a Dectris PILATUS3 S 6M. Data were indexed, integrated and scaled with *DIALS* (Winter *et al.*, 2018 ▸) and imported into the *CCP*4 suite (Agirre *et al.*, 2023 ▸) with *AIMLESS* (Evans & Murshudov, 2013 ▸). The structure was solved with *phenix.mr_rosetta* through a combination of *ab initio* modelling and molecular replacement (DiMaio *et al.*, 2011 ▸; Terwilliger *et al.*, 2012 ▸). Refinement was performed using *phenix.refine* (Afonine *et al.*, 2012 ▸) in conjunction with manual model building in *Coot* (Emsley *et al.*, 2010 ▸). Translation–libration–screw parameters were automatically determined and used by *phenix.refine*. Model geometry was analysed and optimized based on suggestions by *MolProbity* (Williams *et al.*, 2018 ▸). Data-collection and model statistics are summarized in Table 1 ▸.

## 3. Results and discussion

### 3.1. Activity analysis of *Gh*Trp

Several attempts at identifying potential substrates using small chromogenic peptide-based substrates as well as proteomic identification of protease cleavage sites (PICS) analysis against a bacterial (*E. coli*) peptide library (Eckhard *et al.*, 2016 ▸) failed to demonstrate any measurable catalytic activity for *Gh*Trp (data not shown).

### 3.2. Structure solution

A crystallographic property present in the *Gh*Trp crystal structure prevented initial structure solution. Due to the presence of translational noncrystallographic symmetry (tNCS) in the crystal, the structure was unable to be solved using simple molecular-replacement strategies. The tNCS was identified by *phenix.xtriage* (Zwart *et al.*, 2005 ▸), which showed a strong off-origin Patterson peak at (*u*, *v*, *w*) = (0.00, 0.06, −0.50) with a height of 28% of the Patterson origin peak. This structure was solved at a time (early 2021) when structural modelling techniques had yet to reach the more accurate predictive capabilities of *AlphaFold* (Jumper *et al.*, 2021 ▸) and *RosettaFold* (Baek *et al.*, 2021 ▸). The best search model identified through sequence alone only had ∼30% sequence identity and a C^α^ r.m.s.d. of ∼2.5 Å (PDB entry 1dt2), which may have been sufficient for determining phases if not for the artefacts associated with tNCS interfering with molecular-replacement techniques (Read *et al.*, 2013 ▸). This was nevertheless a better search model than the ∼3.0 Å C^α^ r.m.s.d. homology model predicted by *I-TASSER* at that time (Supplementary Fig. S2; Roy *et al.*, 2010 ▸). The structure was ultimately solved using *phenix.mr_rosetta* as this was one of the first programs that incorporated *ab initio* model building as part of the phasing process (DiMaio *et al.*, 2011 ▸; Terwilliger *et al.*, 2012 ▸). As expected, the *Gh*Trp crystal structure depicts two molecules in the asymmetric unit, related to each other along the *c* axis by a tNCS vector of approximately half the *c*-axis length (Supplementary Fig. S3).

### 3.3. The general fold shows modifications to trypsin-like specificity loops

Despite having low sequence identity (<20%) to most known chymotrypsin-like and trypsin-like proteases, *DALI* analysis (Holm, 2022 ▸) demonstrates that the fold of *Gh*Trp is consistent with other members of the S1 family of glutamyl endopeptidases, as categorized by the MEROPS database (Rawlings *et al.*, 2018 ▸). *Gh*Trp exhibits reasonable overall structural homology with this family of glutamyl endopeptidases, with the best-aligning structures having *DALI* scores of >18.5 and overall C^α^ r.m.s.d. values of between 2 and 2.8 Å despite sequence identities of 21% or less (Table 2 ▸).

**Figure 1:**
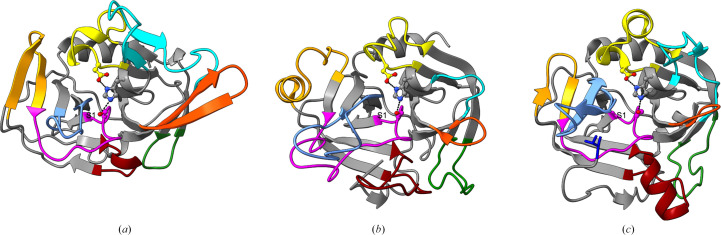
A comparison of the loop structures of (*a*) *Gh*Trp (PDB entry 9ect), (*b*) bovine trypsin (PDB entry 1hj9) and (*c*) *Bacillus intermedius* glutamyl-endopeptidase (PDB entry 1p3c). The known specificity loops, loop A (37 loop; dark orange), loop B (60 loop; cyan), loop C (99 loop; yellow), loop D (148 loop; maroon), loop E (75 loop; green), loop 1 (189 loop; magenta), loop 2 (220 loop; light blue) and loop 3 (175 loop; orange), are illustrated in each structure with the remaining protein rendered in grey. In (*c*), the location of the N-terminus is indicated by the N-terminal leucine residue rendered as a blue stick model. Potential interactions between members of the catalytic triad are rendered as dashed lines and the location of the S1 pocket is annotated. All molecules are presented in an identical orientation.

The trypsin-like fold has been well characterized and the involvement of the many surface loops as determinants of subsite selectivity for peptide and protein substrates has been well documented (reviewed in Goettig *et al.*, 2019 ▸). An analysis of these surface loops in the structure of *Gh*Trp demonstrates that there are considerable differences in the structures of the specificity loops between the classic trypsin structure and *Gh*Trp, with the exception of loop C. Comparisons between the structures of *Gh*Trp, bovine trypsin and *Bacillus intermedius* glutamyl peptidase (BGP; Fig. 1 ▸) demonstrate that loops A and B are considerably larger in bovine trypsin and loops D and E are shorter in *Gh*Trp than either of the other two enzymes. *Gh*Trp therefore lacks the calcium-binding residues that stabilize the more elongated loop structure in bovine trypsin and thus no ions are observed in the structure of *Gh*Trp (Leiros *et al.*, 2001 ▸).

In *Gh*Trp and BGP, loop 3 forms additional β-strands that extend the core β-sheet, which is quite different from the helical structure found in bovine trypsin. Loop 1, which contains the serine nucleophile (Ser167) and the oxyanion-hole residues (amides of Ser167/Gly165), is similar in structure between *Gh*Trp and BGP but is truncated when compared with bovine trypsin. This may be a consequence of their correspondingly truncated loops 2, which act as a supporting structure for the placement of loop 1. As both loops 1 and 2 are truncated in *Gh*Trp relative to bovine trypsin, loop 2 is still able to function as a backing structure for loop 1 in the fold.

**Figure 2:**
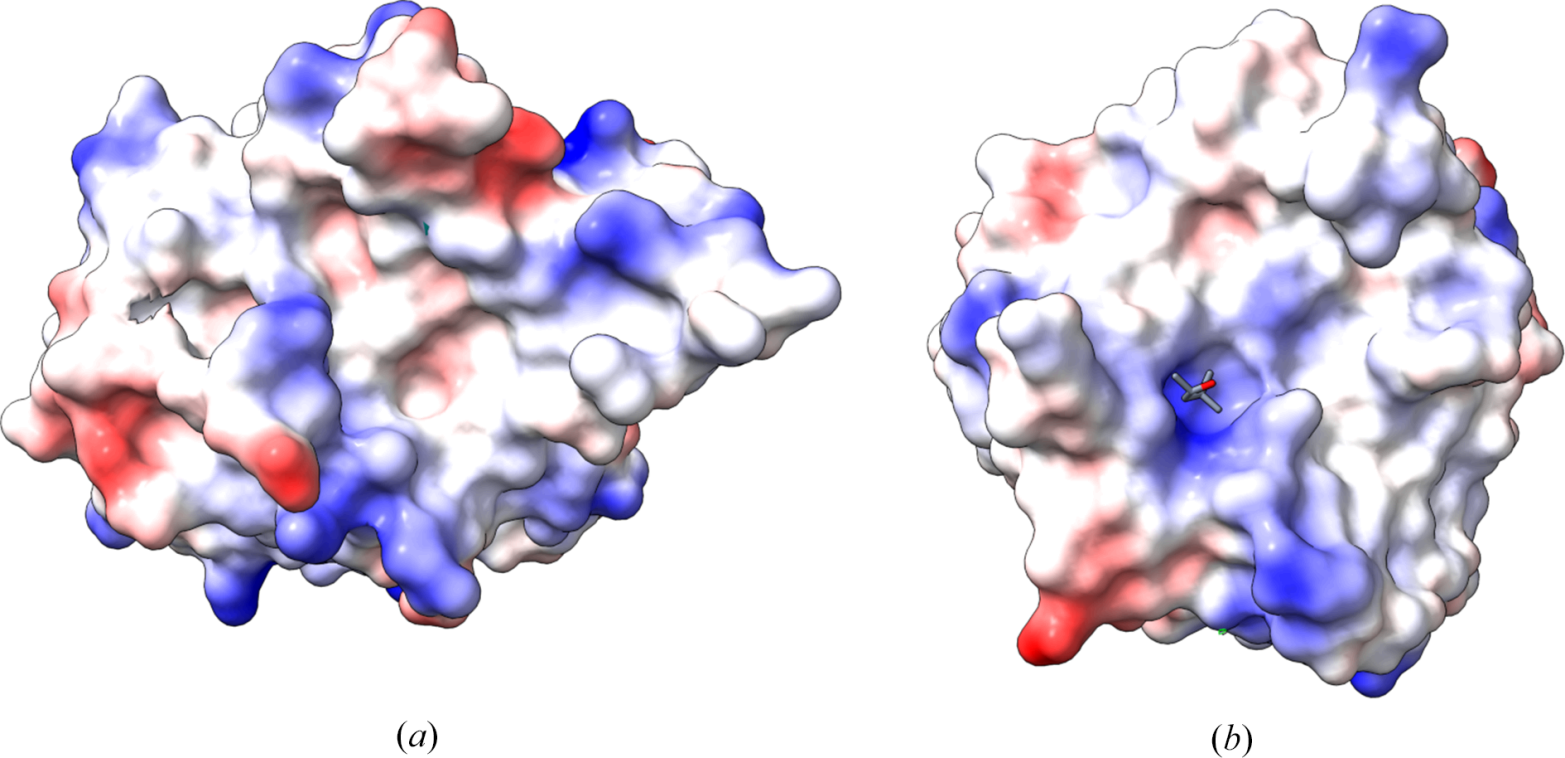
A comparison of the electrostatic surfaces of (*a*) *Gh*Trp (PDB entry 9ect) and (*b*) *B. intermedius* glutamyl-endopeptidase (PDB entry 1p3c). In (*b*) the position of the S1 binding pocket is indicated by the MPD molecule that was co-crystallized (grey sticks coloured by atom type). In the *Gh*Trp structure (*a*), the S1 pocket site is occluded by the structure of loop 2. Both molecules are presented in the same orientation as in Fig. 1 ▸.

Most notably, the conformation of loop 2 of *Gh*Trp places it in the middle of the putative S1 pocket, bifurcating the substrate-binding groove (Fig. 2 ▸). This malformed S1 pocket is consistent with the functional data that demonstrate a lack of proteolytic function for this enzyme construct. In contrast, the the prime-side subsites are well structured. Taken together, these data suggest that the *Gh*Trp structure could represent a pro-enzyme-like form of the putative zymogen in which some activation event is required to properly stabilize loop 2 in an active conformation to generate a viable S1 pocket. One could argue that the bifurcation of the S1 pocket may be the result of characterizing *Gh*Trp outside the context of *Gh*IgAP. However, the *AlphaFold*3 model of full-length *Gh*IgAP shows a similar conformation of loop 2 in which it still bifurcates the S1 pocket, consistent with the persistence of the pro-enzyme-like conformation in the full-length enzyme (Supplementary Fig. S1*b*).

**Figure 3:**
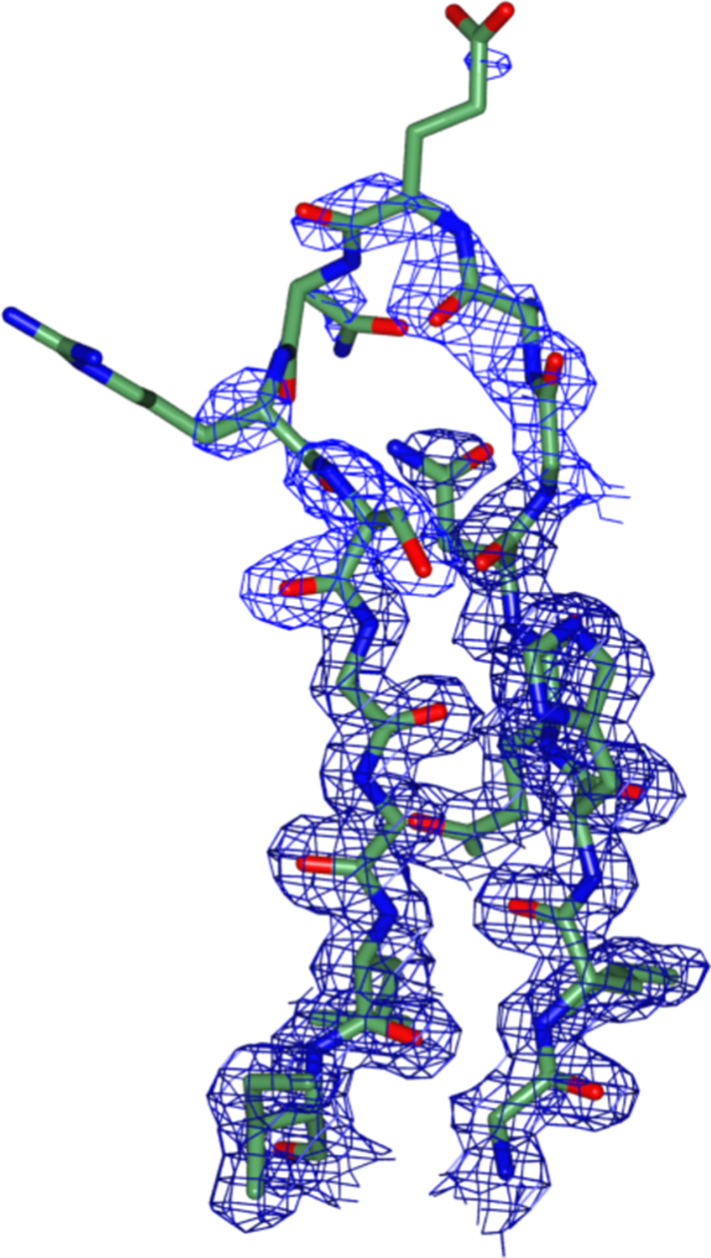
Apparent disorder in loop 2 (863–877) of *Gh*Trp. The backbone and side chains are represented as stick models and coloured by atom type with C atoms in green. 2*F*_o_ − *F*_c_ density at 1σ is rendered as a blue mesh

The N-termini of many trypsin-like serine proteases have been shown to regulate protease activation and activity. For example, the N-terminal helix of the *Staphylococcus aureus* exfoliative toxin A stabilizes the S1 pocket and deletions in the N-terminal region abolish activity (Cavarelli *et al.*, 1997 ▸). An alternative explanation for this substrate-binding-groove bifurcation comes from examining the structure of BGP, where zymogen activation liberating the N-terminal leucine residue stabilizes a correct loop 2 conformation and formation of the S1 pocket (Fig. 1 ▸*c*; Meijers *et al.*, 2004 ▸). In the crystallized construct, the N-terminus of *Gh*Trp is too short to interact with loop 1 to stabilize an open, active conformation. Even if the N-terminus is extended by ∼30 amino acids, *Gh*Trp remained inactive and this extra N-terminal tail was shown to lack a defined structure (Redzic *et al.*, 2022 ▸). If the *Gh*Trp structure truly depicts a pro-enzyme, the activation mechanism for BGP suggests that the N-terminus of *Gh*Trp must be cleaved at a specific site to properly activate *Gh*Trp. Further support for this activation mechanism comes from the electron-density maps corresponding to loop 2, in which the distal end of this loop (residues 868–872) is poorly ordered in the crystal structure in its modelled conformation (Fig. 3 ▸). However, based upon the data, we cannot rule out the possibility that this domain of *Gh*IgAP evolved from the trypsin protease fold, lost its ability to function as a protease and acquired a different, but as of yet unknown, function.

## 4. Conclusions

The crystal structure of *Gh*Trp was solved to gain insight into the potential functions of this domain despite difficulties in finding a substrate for the putative enzyme. These structural data showed that the lack of activity observed is unsurprising due to the aberrant position of loop 2 occluding the S1 pocket in the crystal structure and *AlphaFold* model. Based upon this result, we hypothesize that the current structure of *Gh*Trp represents the pro-enzyme structure of the enzyme that is present in the full-length M26 IgAP. We hypothesize that this putative pro-enzyme form must undergo a specific cleavage event to generate an N-terminal segment that interacts with loop 2 to stabilize a more open and active conformation of the S1 pocket.

## Supplementary Material

PDB reference: *Gemella haemolysans* M26 IgA1 protease trypsin-like domain, 9ect

Supplementary Figures. DOI: 10.1107/S2053230X25001219/no5210sup1.pdf

## Figures and Tables

**Table 1:** Data-collection and refinement statistics for *Gh*Trp (PDB entry 9ect) Values in parentheses are for the highest resolution shell.

Wavelength (Å)	1.521
Resolution range (Å)	74.21–1.75 (1.79–1.75)
Space group	*P*2_1_
*a*, *b*, *c* (Å)	47.27, 58.55, 76.37
α, β, γ (°)	90, 103.66, 90
Total reflections	233882 (21006)
Unique reflections	40706 (4006)
Multiplicity	5.7 (5.2)
Completeness (%)	99.30 (98.86)
Mean *I*/σ(*I*)	2.60 (0.28)
Wilson *B* factor (Å^2^)	13.03
*R*_merge_	0.205 (0.621)
*R*_meas_	0.224 (0.691)
*R*_p.i.m._	0.089 (0.296)
CC_1/2_	0.984 (0.835)
No. of reflections used in refinement	40700 (4005)
No. of reflections used for *R*_free_	2031 (181)
*R*_work_	0.1936 (0.2865)
*R*_free_	0.2421 (0.3443)
No. of atoms	
Total	3855
Protein	3313
Water	542
*B* factors (Å^2^)	
Overall	20.94
Protein	19.67
Water	28.67
Root-mean-square deviations	
Bond lengths (Å)	0.004
Angles (°)	0.735
Rotamer outliers (%)	0
Clashscore	3.65
Ramachandran statistics (%)	
Favoured	98.32
Allowed	1.68
Outliers	0

**Table 2:** Top-ranking structures from *DALI* analysis of *Gh*Trp against the PDB50 data set

Protein	PDB code	*DALIZ*-score	C^α^ r.m.s.d. (Å)	Sequence identity (%)
*Bacillus intermedius* glutamyl-endopeptidase	1p3c	21.8	2.0	19
*Arthrobacter nicotinovorans* protease	3wy8	19.6	2.4	12
Protease DO	4ynn	19.3	2.8	21
Exfoliative toxin D2	5c2z	19.1	2.3	21
Exfoliative toxin C	8r3i	18.9	2.3	21
Epidermolytic toxin A	1agj	18.6	2.2	21

## Data Availability

The model coordinates and structure factors for *Gh*Trp have been deposited in the PDB (https://www.rcsb.org/pdb) under accession code 9ect. The PDB deposition is cross-referenced with residues 661–873 of UniProt entry C5NYF3. It should be noted that this entry contains a sequence that lacks 23 N-terminal residues relative to the *Gh*IgAP sequence used in this and previous literature (GenBank WP_040464465.1; residues 684–896).

## Footnotes

1 To be consistent with the numbering found in the deposited full-length structure of *Gh*IgAP (PDB entry 7uvk), in the manuscript we have used the sequence numbering corresponding to NCBI entry WP_040464465.1, which is offset from the sequence found in the PDB deposition corresponding to UniProt entry C5NYF3 by 23 residues.
