# Supplementary material for: The structure of the Gemella haemolysans M26 IgA1 protease trypsin-like domain
Source: Acta Crystallogr F Struct Biol Commun. 2025 Feb 28;81(Pt 4):124–9. doi: 10.1107/S2053230X25001219 (PMC11970125; doi:10.1107/S2053230X25001219)
Supplement: Supplementary file 1 [file f-81-00124-sup1.pdf]

STRUCTURAL BIOLOGY
COMMUNICATIONS

Volume 81 (2025)

Supporting information for article:

The structure of the *Gemella haemolysans* M26 IgA1 protease trypsin-like domain

Norman Tran, Jasmina S. Redzic, Elan Z. Eisenmesser and Todd Holyoak

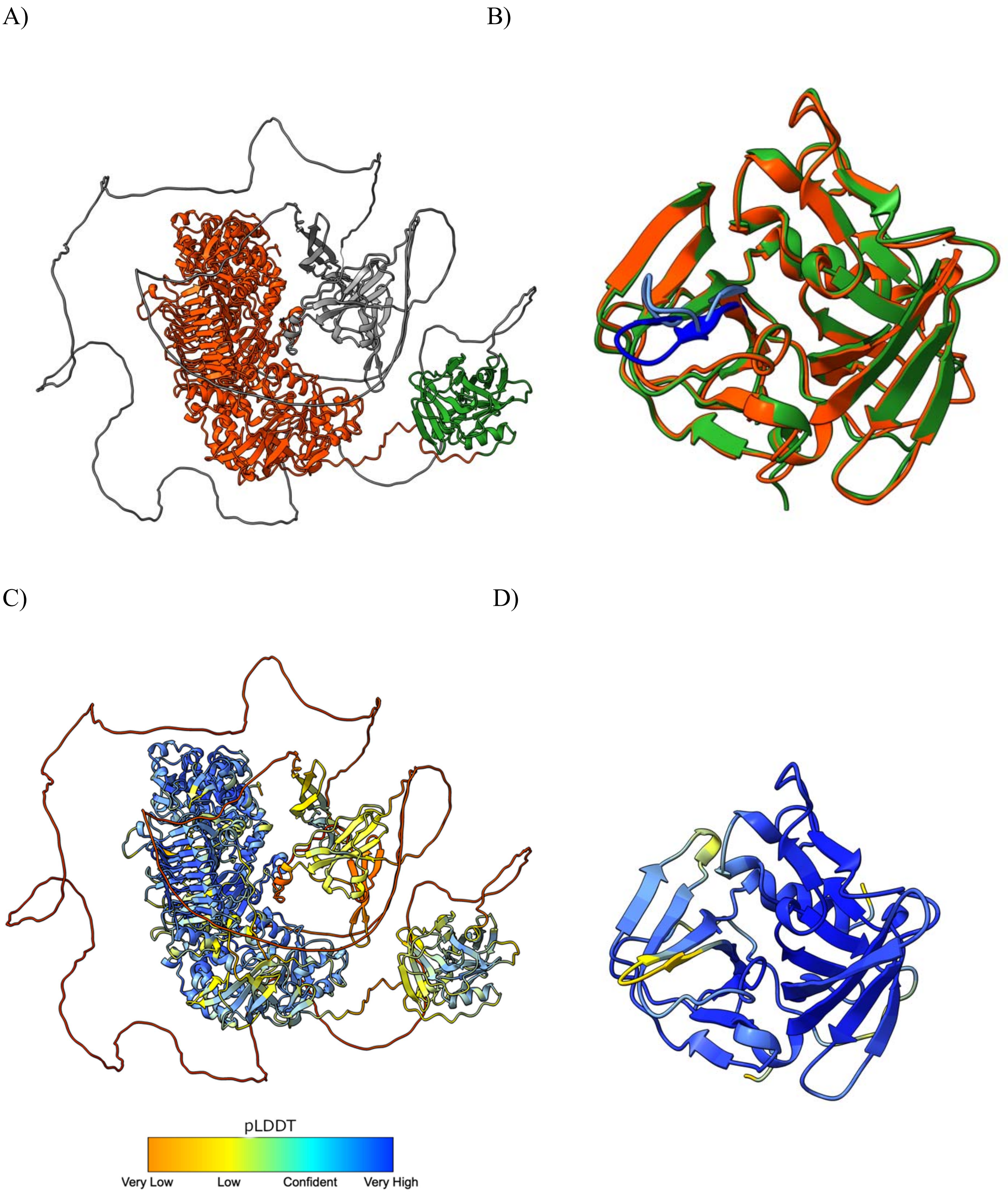

Figure S1 A comparison of the AlphaFold3-predicted model of *GhIgAP* with the *GhTrp* crystal structure. The Google webserver (https://alphafoldserver.com) for AlphaFold3 was used to generate the full-length model using default settings.A) the complete modelled structure of *GhIgAP* with the IgAP domain rendered in orange and the trypsin domain in green. The remainder of the N-terminal structure is rendered in grey. B) superimposition of the *GhTrp* domain from the AlphaFold model (green) with the crystallographic structure (orange). Loop 2 in the modelled and crystallographic structures is rendered in in dark and light blue, respectively. The two structures align well with a Cα RMSD of 1.4Å. The complete GhIgAP (C) and the GhTrp domain (D) AlphaFold3 predicted

structures colored by pLDDT values. The color key corresponds to Very high (pLDDT > 90), Confident (90 > pLDDT > 70), Low (70 > pLDDT > 50), Very low (pLDDT < 50).

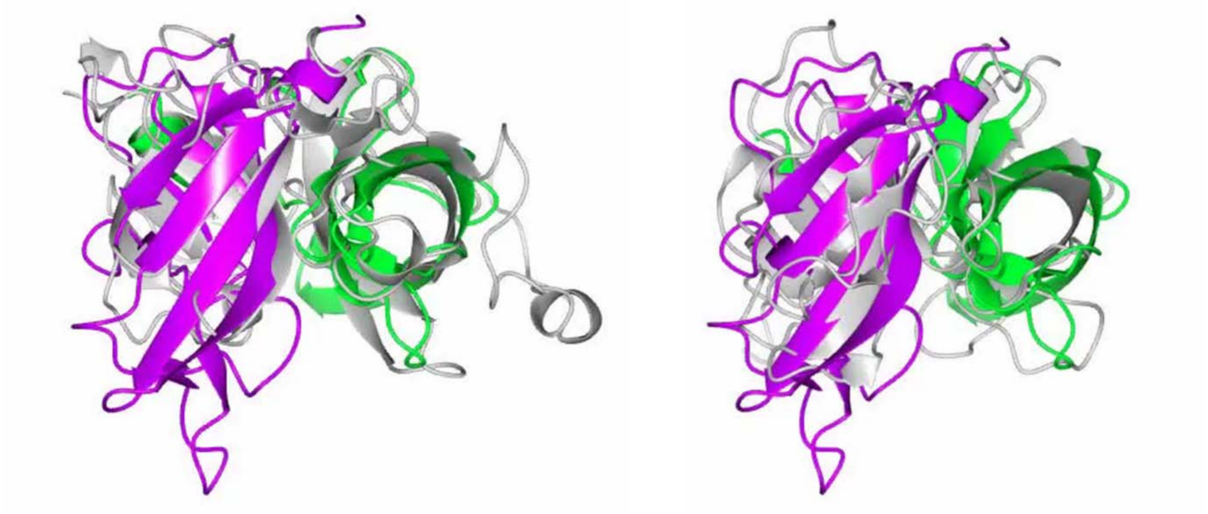

A) B)

Figure S2 Superimposition of *GhTrp* with previously unsuccessful molecular-replacement search models. The N-terminal (purple) and C-terminal (green) lobes of the *GhTrp* trypsin-like fold are superimposed with A) the highest sequence identity model at the time (31.2% identity; Cα RMSD of 2.46 Å) and B) the I-TASSER homology model (Cα RMSD of 3.03 Å). These search models are shown in gray.

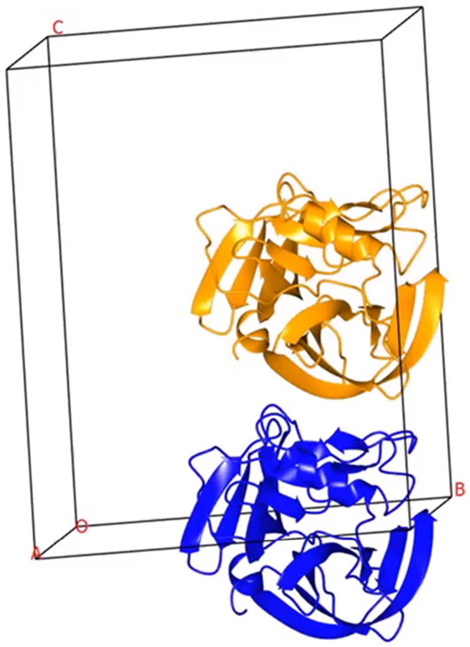

Figure S3 Asymmetric unit of the *GhTrp* in the context of the unit cell. The two molecules of the *GhTrp* asymmetric unit are shown in blue and orange. They are related to each other by translational non-crystallographic symmetry along the c-axis of the unit cell.
